# Clinical Applications and Future Directions of Smartphone Fundus Imaging

**DOI:** 10.3390/diagnostics14131395

**Published:** 2024-06-30

**Authors:** Seong Joon Ahn, Young Hwan Kim

**Affiliations:** Department of Ophthalmology, Hanyang University Hospital, Hanyang University College of Medicine, Seoul 04763, Republic of Korea

**Keywords:** smartphone, fundus imaging, clinical applications, future directions

## Abstract

The advent of smartphone fundus imaging technology has marked a significant evolution in the field of ophthalmology, offering a novel approach to the diagnosis and management of retinopathy. This review provides an overview of smartphone fundus imaging, including clinical applications, advantages, limitations, clinical applications, and future directions. The traditional fundus imaging techniques are limited by their cost, portability, and accessibility, particularly in resource-limited settings. Smartphone fundus imaging emerges as a cost-effective, portable, and accessible alternative. This technology facilitates the early detection and monitoring of various retinal pathologies, including diabetic retinopathy, age-related macular degeneration, and retinal vascular disorders, thereby democratizing access to essential diagnostic services. Despite its advantages, smartphone fundus imaging faces challenges in image quality, standardization, regulatory considerations, and medicolegal issues. By addressing these limitations, this review highlights the areas for future research and development to fully harness the potential of smartphone fundus imaging in enhancing patient care and visual outcomes. The integration of this technology into telemedicine is also discussed, underscoring its role in facilitating remote patient care and collaborative care among physicians. Through this review, we aim to contribute to the understanding and advancement of smartphone fundus imaging as a valuable tool in ophthalmic practice, paving the way for its broader adoption and integration into medical diagnostics.

## 1. Introduction

Since Jackman and Webster reported retinal photographs in 1886, fundus imaging plays a crucial role in ophthalmology by providing the detailed visualization of the retina, optic nerve head, and surrounding structures, aiding in the diagnosis and management of various ocular conditions [1]. Traditional fundus imaging techniques, which primarily rely on specialized cameras and equipment, have long been the gold standard for capturing high-quality retinal images [1,2]. However, these conventional methods have limitations, including limited portability and restricted accessibility, particularly in resource-limited settings [3].

Recently, the advent of smartphone technology has revolutionized the field of fundus imaging, offering a promising solution to address the shortcomings of the traditional approaches [3]. Smartphone fundus imaging involves the use of smartphones equipped with or without specially designed attachments or accessories to capture retinal images [4,5,6,7]. The optics involved in smartphone fundus imaging typically include condensing lenses or adapters that attach to the smartphone camera, facilitating the visualization of the fundus [1,3,8,9]. These accessories optimize the optical pathway, allowing for the acquisition of retinal images directly through the smartphone’s camera lens [10]. This innovative approach has led to the development and evolution of smartphone fundus imaging technology, establishing it as a low-cost, portable, and accessible alternative to traditional fundus imaging modalities, such as fundus photography and fluorescein angiography [3].

The importance of smartphone fundus imaging in clinical practice cannot be overstated. For example, its low cost, portability, and ease of image acquisition have democratized access to retinal imaging, enabling the earlier detection, diagnosis, and monitoring of various retinal pathologies [3,5,10,11,12]. Moreover, smartphone fundus imaging has the potential to revolutionize telemedicine and remote patient monitoring, particularly in underserved areas and low-resource settings where access to specialized ophthalmic care is limited [13]. Additionally, integrating smartphone fundus imaging into primary care settings holds promise for enhancing the early detection of sight-threatening conditions and facilitating timely referrals to ophthalmic specialists [14]. 

There have been several efforts to apply this technique to various ophthalmic diseases [3,5,10,11,12,13]. However, this has not been outlined over diverse retinal diseases, as techniques and specific indications have been mainly addressed in previous research and review papers (Table S1) [3,10]. Therefore, this review paper aims to fill this gap by providing a comprehensive analysis of the clinical applications, both conventional and novel ones, and future directions of smartphone fundus imaging in retinal diseases, together with an overview of smartphone fundus imaging, including its advantages, limitations, and techniques. From the existing literature and evidence, we aimed to elucidate the potential of smartphone fundus imaging as a valuable tool that can be more widely used in the practice of managing retinal diseases. Furthermore, we intended to outline areas for future investigation in this rapidly evolving field. 

## 2. Advantages and Limitations

Table 1 summarizes the advantages and limitations of current smartphone fundus imaging. 

### 2.1. Advantages

Smartphone fundus imaging offers several significant advantages over traditional methods, particularly in terms of accessibility and portability [3,5,11,12]. The use of smartphones for capturing retinal images makes the technology extremely portable and accessible, allowing healthcare providers to perform fundus examinations in a variety of settings, including remote or rural areas where traditional fundus cameras are not feasible. This portability also facilitates the use of these devices in non-traditional environments such as emergency rooms, nursing homes, or in patients’ homes, providing a critical tool for immediate assessment [15]. Additionally, despite ethical considerations, the ease of sharing images through smartphones can significantly enhance collaborative care, enabling quick consultations with specialists or other healthcare providers without the need for the physical transfer of patients or traditional imaging files [16].

Cost-effectiveness is another compelling advantage of smartphone fundus imaging. Smartphone fundus imaging offers a significant reduction in equipment costs compared to traditional fundus cameras. Traditional fundus cameras, especially those that are table-top models, can be prohibitively expensive, often costing upwards of USD 10,000, and often require additional costs for maintenance and training [17]. In contrast, smartphones are widely available and relatively inexpensive, with some setups costing below USD 1000 [17]. This makes them accessible to a broader range of healthcare providers, particularly in low- and middle-income countries. This dramatic reduction in price makes it feasible to equip more healthcare providers with fundus imaging capabilities, thereby expanding access to essential diagnostic services. The lower cost also opens up possibilities for widespread screening programs, particularly in low-resource settings where the financial burden of traditional equipment has been a barrier [18].

Moreover, only a slight decrease in accuracy observed with smartphone-based methods (e.g., sensitivity and specificity in detecting diabetic retinopathy [DR] and sight-threatening DR) is often within an acceptable range for clinical use. For instance, a study reported a sensitivity of 75.2% and specificity of 95.2% for detecting any DR with a smartphone-based non-mydriatic camera compared to conventional mydriatic fundus cameras [19]. Another study showed the reliability of an offline AI algorithm for fundus images captured by non-specialist operators using a smartphone-based camera, with a sensitivity of 89.1% and specificity of 94.4% for any DR [20]. 

These figures suggest that despite some (usually less than 25%) loss of accuracy, smartphone-based methods still provide robust diagnostic capabilities and thus, decreasing 90% percent equipment cost for hospitals and the per-use cost for patients while only decreasing less than 25% accuracy may particularly attract doctors to use the smartphone methods. 

Furthermore, the integration of machine learning algorithms and artificial intelligence with smartphone fundus imaging enhances its diagnostic capabilities. These technologies can help in the automated grading of fundus images, further reducing the need for specialist interpretation and making the process more efficient [3]. This integration can significantly improve the scalability of screening programs, ensuring the timely diagnosis and management of retinal conditions.

The integration of smartphone technology into fundus imaging can also enhance patient engagement and empowerment. By utilizing a device that is familiar and readily available, patients may feel more comfortable and involved in their diagnostic processes [4,21]. The ability to immediately view and discuss the images taken can help in educating patients about their health conditions, potentially increasing their engagement in managing their health. This aspect of patient empowerment is crucial, especially in chronic conditions like diabetes, where ongoing monitoring and management are key to preventing vision-threatening complications. Moreover, the adaptability of smartphones allows for continuous improvements and updates in software and hardware. As smartphone technology advances, the quality of images and the functionality of imaging apps can improve, often without the need to purchase new and expensive equipment [22]. 

### 2.2. Limitations

Smartphone fundus imaging, while innovative and accessible, presents several limitations that can impact its clinical utility, particularly concerning image quality and resolution [23]. Despite significant improvements over the years, the optical setup of smartphone cameras still falls short of the high standards set by traditional fundus cameras. The resolution of the images captured via smartphones may not always be sufficient for detailed diagnosis, especially for detecting subtle retinal changes. Additionally, the field of view is often limited compared to traditional devices, typically capturing a smaller area of the retina in a single image [3]. This limitation, potentially leading to missing critical peripheral retinal details, can necessitate multiple images, adjustments during examination, or the use of additional adaptors for wide-field imaging [4].

Conventional digital fundus cameras typically feature image sensors with varying pixel counts, often around 12 megapixels (MP) [24]. Modern smartphone cameras may also have high pixel counts; for instance, the study using an iPhone 6 for fundus imaging had an image resolution of 2448 × 3264 pixels (about 8 MP) for single-image acquisition and 1248 × 1664 pixels (about 2 MP) for video mode [23]. However, it is important to note that more pixels do not necessarily translate to better image quality for fundus photography. Due to the optical limitations of the eye itself, there is a point beyond which additional pixels do not improve resolution. The key factors for image quality in fundus photography are often the optics of the system and the eye being imaged, rather than just raw pixel count. Therefore, smartphone-based systems might produce clinically useful images despite potentially lower effective resolutions compared to high-end conventional fundus cameras.

Another significant challenge with smartphone fundus imaging is the lack of standardization and calibration across different devices [25]. Unlike traditional fundus cameras, which follow strict manufacturing and performance standards, smartphone-based systems can vary widely depending on the phone model, camera quality, and additional optical attachments used. This variability can lead to inconsistencies in image quality and diagnostic accuracy, making it difficult to compare images across different setups or to establish reliable baselines for patient monitoring over time. The absence of standardized calibration procedures for these setups further complicates their use in clinical practice, where precise measurements and reproducibility are crucial.

Regulatory considerations also pose a notable limitation for the widespread adoption of smartphone fundus imaging [26]. Medical devices, particularly those used for diagnostic purposes, are subject to stringent regulatory standards to ensure safety and efficacy. Smartphone-based imaging systems often straddle the line between consumer electronics and medical devices, leading to potential challenges in meeting regulatory requirements. This situation is complicated by the rapid pace of technological change in consumer electronics, which can outstrip the slower regulatory processes [25].

Medicolegal issues related to the easy sharing of images are another concern. While the ability to share retinal images easily can enhance collaborative care and consultation, it also raises significant concerns about patient privacy and data security [27]. The ease with which digital images can be transferred and stored on various platforms necessitates robust safeguards to protect sensitive health information. Without proper security measures, there is a risk of unauthorized access and potential breaches of confidentiality, which could have serious medicolegal implications for healthcare providers [28].

In summary, while smartphone fundus imaging offers several advantages in terms of accessibility and cost, its limitations related to image quality, the lack of standardization, regulatory challenges, and medicolegal risks must be carefully considered. These issues highlight the need for ongoing research, development, and, perhaps most importantly, a regulatory framework that can keep pace with technological advancements while ensuring patient safety and the reliability of this technology in medical diagnostics.

## 3. Techniques of Smartphone Fundus Imaging

### 3.1. Techniques Used and Tips

**Figure 1:**
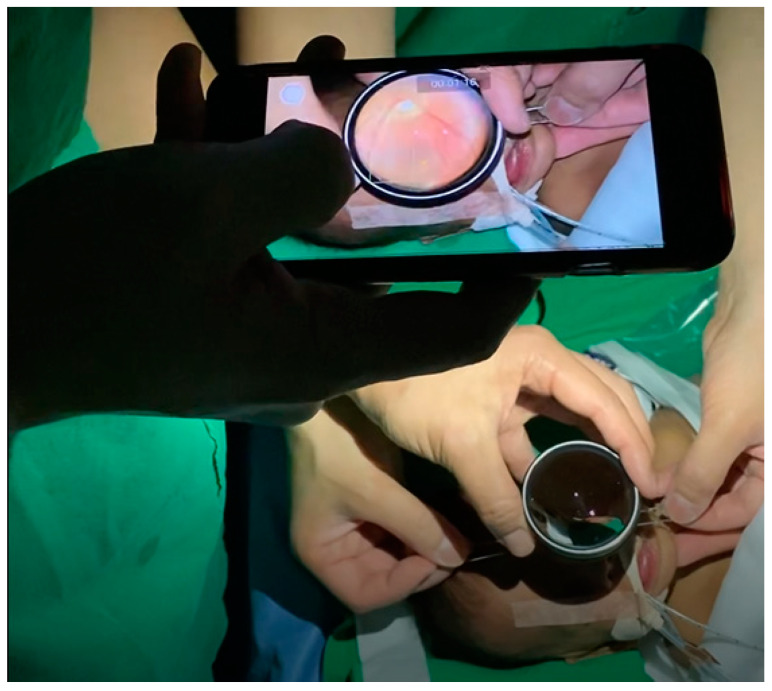
Technique of smartphone fundus imaging for the evaluation of the retinopathy of prematurity.

Smartphone fundus imaging, with or without associated accessories, utilizes the optical capabilities of smartphones to capture retinal images (Figure 1) and encompasses various techniques and methodologies for capturing high-quality retinal images [29]. Here is an overview of the general process involved in smartphone fundus imaging:

Pupil Dilation: Prior to imaging, the patient’s pupil is dilated using topical ophthalmic drops to optimize the visualization of the fundus. While pupil dilation is not always strictly required for smartphone fundus imaging, it significantly improves the image quality [30].

Preparation of Smartphone: The smartphone camera is set to video mode, allowing for the recording of live images. The camera’s flashlight is turned on to provide illumination during imaging.

Positioning of Optical Lens and Smartphone: A condensing lens, a handheld +15–+30 D lens, is positioned approximately 3–5 cm in front of the patient’s eye. The smartphone camera is held at a distance of 10–35 cm from the lens attachment. The camera and lens are adjusted to achieve optimal focus and minimize light reflections.

Image Capture: Once a clear view of the fundus is obtained, the camera record button is pressed to begin capturing the retinal images as a video. The examiner may move the camera and lens slightly to explore the different areas of the retina and obtain comprehensive images.

Image Review and Documentation: After recording the video, specific frames containing clear fundus images are selected for further analysis. Screenshots or still images are captured from the video frames to document the findings. Additional image processing or enhancement may be performed using smartphone apps or software tools.

**Figure 2:**
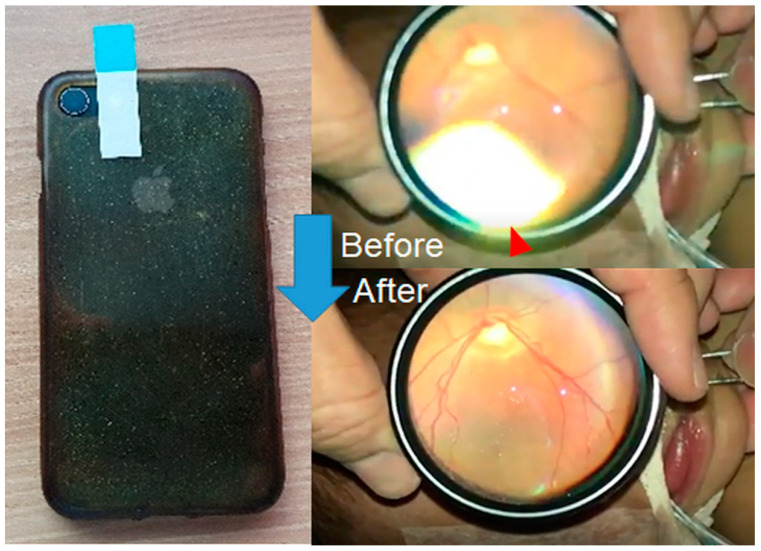
Technique of reducing light reflection using semi-transparent tape. After attaching the semi-transparent tape to the flashlight, the light reflection (red arrowhead) is remarkably reduced.

By following these steps, healthcare providers can effectively capture high-quality retinal images using smartphones for diagnostic and monitoring purposes in various clinical settings. However, some tips may facilitate the image acquisition by smartphone fundus imaging. Here are a few tips:

Reduce light reflection by using semi-transparent tape: Attach a tape to the light source to diminish the intensity of the flashlight (camera flash, Figure 2).

Complete Smartphone Settings Followed by Lens Positioning: Set up the smartphone screen to magnify the patient’s eye on the screen, then position the lens in front of the patient’s eye. Setting them one by one is easier than doing both simultaneously.

### 3.2. Methods Using Devices or Software

The use of adapters for smartphone fundus imaging has significantly enhanced the practicality and quality of retinal photography [31]. These adapters are designed to hold a condensing lens at a precise, adjustable distance from the smartphone camera, ensuring optimal alignment and focus [31]. This setup minimizes the guesswork involved in manually estimating distances, thereby facilitating the capture of high-quality, reproducible images. For instance, 3D-printed mounts can securely attach lenses like the Pan Retinal 2.2 BIO Lens (Volk Optical, Inc., Mentor, OH, USA) to smartphones, allowing for stable and consistent imaging. Additionally, devices such as the iExaminer (WelchAllyn, Skaneateles Falls, NY, USA) and D-Eye adapter (D-EYE Srl, Padova, Italy) offer integrated solutions that combine the smartphone with specialized ophthalmoscopes, further simplifying the imaging process and improving the field of view and image quality. These advancements make smartphone fundus imaging a viable, low-cost alternative to traditional fundus cameras, particularly beneficial in low-resource settings.

By incorporating these methods and tools, smartphone fundus imaging can become a more reliable and efficient approach for capturing retinal images, enhancing its utility in diverse healthcare environments.

## 4. Clinical Applications of Smartphone Fundus Imaging

### 4.1. Screening and Diagnosis of Retinal Diseases

Smartphone fundus imaging has emerged as a pivotal tool in the screening and diagnosis of various retinal diseases leveraging the widespread availability and advancing capabilities of smartphone technology. The expanding role of smartphone fundus imaging in clinical settings underscores its potential to enhance the accessibility and efficiency of retinal disease screening [11,32,33,34]. The benefits of smartphone fundus imaging, particularly in resource-limited settings and for conditions requiring immediate attention, make it a valuable addition to the ophthalmic diagnostic toolkit [11]. Table 2 summarizes the results of the application of smartphone fundus imaging for screening or diagnosing retinal diseases. 

#### 4.1.1. Diabetic Retinopathy

DR is one of the primary applications of smartphone fundus imaging, especially in settings where access to traditional ophthalmic equipment is limited. Studies have demonstrated that smartphone fundus imaging can effectively screen for DR by capturing detailed images of the retina, allowing for the identification of typical DR changes such as microaneurysms, hemorrhages, and exudates [6,11,33,34,35]. The sensitivity and specificity of these devices can vary, but some studies report sensitivity as high as 91% and specificity up to 99% for detecting any DR, making it a viable option for preliminary screening [6,11,33,34,35].

More specifically, Ryan et al. evaluated the diagnostic capability of smartphone imaging, finding a sensitivity of 50% and specificity of 94%, which improved to 81% and 94%, respectively, with a non-mydriatic fundus camera [33]. Russo et al. focused on clinically significant macular edema, reporting a sensitivity of 81% and specificity of 98% [34]. Toy et al. examined moderate nonproliferative and worse DR, achieving 91% sensitivity and 99% specificity [6]. Wintergerst et al. reported a sensitivity of 0.79 and specificity of 0.99 for any DR, 1.0 for both measures in severe DR, and 0.79 sensitivity and 1.0 specificity for diabetic maculopathy [11]. Recently, Gobbi et al. found a sensitivity of 71% and specificity of 94% for DR detection [35]. 

However, the portability of smartphones enables frequent monitoring, crucial for managing this progressive disease, which is especially useful in rural or underserved areas.

#### 4.1.2. Retinopathy of Prematurity

In the context of the retinopathy of prematurity (ROP), smartphone fundus imaging offers a unique advantage in monitoring the presence or progression of ROP in preterm infants, where transportation to a specialized facility can be risky. The use of smartphone fundus imaging allows for bedside imaging, reducing the stress on the infant and providing essential diagnostic information. This application is particularly valuable in neonatal intensive care units, enabling bedside fundus photography and telemedicine through image sharing. 

Accordingly, several studies reported the clinical utility of smartphone fundus imaging for the evaluation of ROP. Goyal et al. demonstrated that wide-field smartphone imaging produced good image quality in 89.3% of the cases, underscoring its potential for high-quality retinal imaging [8]. Wintergerst et al. compared non-contact smartphone-based fundus imaging with conventional contact fundus imaging and found high diagnostic accuracy, with sensitivities of 90% and 88% and specificities of 100% and 93% for detecting plus disease and ROP, respectively [12]. Patel et al. reported substantial agreement between smartphone-based photographic assessment and the gold standard of indirect ophthalmoscopy in detecting plus disease with a Cohen’s kappa of 0.85, indicating high reliability [36]. Lin et al. showed moderate agreement between binocular indirect ophthalmoscopy and smartphone-based imaging with a Cohen’s kappa of 0.619, highlighting the potential but also areas for the improvement of smartphone imaging [37]. 

#### 4.1.3. Age-Related Macular Degeneration

Age-related macular degeneration (AMD) is another condition where smartphone fundus imaging can play a diagnostic role. While the detailed imaging required to identify early AMD changes might be beyond some current smartphone fundus imaging capabilities (OCT is necessary for the detection of such changes), it is sufficiently accurate for detecting changes associated with advanced stages, such as geographic atrophy and subretinal hemorrhage. As technology advances, the potential for the earlier detection and monitoring of AMD using smartphone fundus imaging in primary care settings could significantly aid in managing the condition at earlier stages. For example, Sayadia et al. present an automated method for the real-time screening of AMD using fundus images on mobile devices [38]. The proposed system leverages advanced image processing techniques and machine learning algorithms to facilitate rapid and accurate AMD detection, with an accuracy ranging from 94% to 100%. This enables early diagnosis and treatment, potentially reducing the incidence of severe vision loss [38].

#### 4.1.4. Retinal Vascular Disorders

Smartphone fundus imaging can be applied in the diagnosis and management of acute retinal vascular disorders, such as retinal vein occlusion (RVO) and retinal artery occlusion (RAO). RAO, which can lead to sudden visual loss, requires rapid diagnosis and intervention. The ability of smartphone fundus imaging to be used on-site by non-specialists allows for immediate imaging and potential early detection, which is crucial for timely referral and treatment. The quality of the images obtained can be sufficient to identify hallmark signs, such as retinal hemorrhages over the wedge-shaped area in branch RVO and retinal whitening in RAO [40].

#### 4.1.5. Hypertensive Retinopathy

The study by Muiesan et al. investigates the feasibility and reliability of using a smartphone-based device (D-Eye) for ocular fundus photography in an emergency department setting for patients with acute hypertension. The results show that the smartphone device significantly detected more abnormal ocular fundus findings compared to traditional ophthalmoscopy, suggesting its potential utility in an emergency department for a better diagnosis of hypertensive emergencies [41].

#### 4.1.6. Other Miscellaneous

Retinoblastoma can be detected using smartphone retinal imaging. As early detection is crucial for effective treatment in patients with this disease, typically young children, smartphone imaging provides a portable and accessible means for early screening [4].

Ocular Toxoplasmosis, caused by the *Toxoplasma gondii* parasite, can lead to retinal inflammation and scarring as well as vitritis, leading to significant visual decline. Recurrence is relatively common, which requires regular monitoring and surveillance of the patients. Smartphone retinal imaging may be useful to document and monitor the retinal changes associated with this condition, particularly the early detection of recurrence [42]. 

### 4.2. Monitoring Disease Progression and Treatment Response

Smartphone fundus imaging has emerged as a pivotal tool in the monitoring and management of DR, a major cause of vision impairment among diabetic patients. This technology allows for the frequent and cost-effective screening of the retina, enabling the early detection of diabetic changes that may require intervention. The portability and ease of use of smartphone-based cameras facilitate regular follow-ups, essential for observing the progression of DR. Studies have demonstrated that smartphone fundus imaging can achieve high sensitivity and specificity in detecting DR, making it a reliable alternative to traditional ophthalmoscopic methods in various clinical settings [11].

In the context of ROP, smartphone fundus imaging serves as a crucial monitoring tool, particularly in neonatal care units where conventional imaging systems may be impractical. The ability to use smartphones for fundus imaging allows for immediate assessment and timely treatment decisions, which are critical in preventing potential blindness in premature infants. The technology’s adaptability for use in pediatric settings, combined with its non-invasive nature, makes it particularly valuable for the continuous monitoring of ROP progression and treatment efficacy. This application is especially beneficial in resource-limited settings where access to specialized ophthalmic equipment and expertise is often restricted.

Moreover, smartphone fundus imaging is instrumental in the management of other retinal conditions such as retinal vein occlusions and age-related macular degeneration. By providing a means to document and monitor subtle retinal changes, such as retinal neovascularization and small retinal hemorrhages, this technology supports the adjustment of therapeutic strategies accordingly. The integration of smartphone imaging into clinical practice not only enhances patient care by facilitating more dynamic management plans but also contributes to the broader field of teleophthalmology, where images can be shared and reviewed by specialists remotely, ensuring expert evaluation and guidance regardless of geographical barriers.

### 4.3. Telemedicine

Smartphone fundus imaging has significantly enhanced the capabilities of telemedicine in ophthalmology, particularly in remote diagnosis, consultation, and second opinions. This technology allows for the capture of high-quality images of the retina using a smartphone equipped with a specialized adapter and lens, making it possible to conduct detailed examinations of the eye from remote locations. The images captured can be transmitted instantly to specialists anywhere in the world, facilitating rapid diagnosis and expert consultation. This is particularly valuable in rural or underserved areas where access to ophthalmologists is limited. The ability to share images securely and discuss cases in real-time with other healthcare professionals helps in making more accurate diagnoses and planning appropriate management strategies for various ocular diseases, such as DR and glaucoma [43,44,45,46].

In addition to aiding in diagnosis and consultation, smartphone fundus imaging plays a crucial role in the follow-up and monitoring of patients with chronic eye conditions [21]. Regular monitoring is essential for conditions like DR, where changes in the retina can occur rapidly and require timely intervention to prevent vision loss. By using smartphone-based fundus cameras, healthcare providers can perform follow-ups more frequently and conveniently without requiring patients to visit a clinic. This not only saves time and reduces travel expenses for patients but also ensures the continuous monitoring of their condition. The images obtained can be compared over time to detect any changes, allowing for dynamic adjustments to treatment plans based on the patient’s current condition or progression.

Furthermore, the integration of smartphone fundus imaging into telemedicine has facilitated educational opportunities and collaborative care approaches. Medical students and trainees in ophthalmology can gain valuable experience by reviewing and discussing diverse retinal images shared by their mentors or peers through telemedicine platforms. This educational aspect is enhanced by the technology’s ease of use and the ability to obtain high-quality images that can be used for teaching and demonstration purposes. Additionally, in complex cases, the availability of remote consultations with retinal specialists can enhance patient care by combining expertise from multiple clinicians, thereby improving outcomes for patients with serious retinal conditions.

### 4.4. Novel Clinical Applications

#### 4.4.1. Pre-, Intra-, or Post-surgical Confirmation of Fundus in the Operating Room

**Figure 3:**
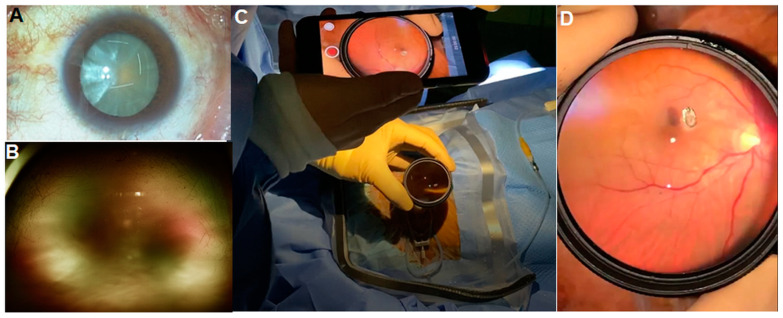
Immediate postoperative fundus imaging in the operating room following a cataract surgery for an eye with a dense cataract (**A**) in which the fundus could not be visualized in ultra-widefield fundus photography preoperatively (**B**). After successful cataract surgery, smartphone fundus imaging (**C**) revealed the details of the posterior pole, including the optic disc and macula (**D**).

Smartphone fundus imaging offers a versatile and portable solution for preoperative and postoperative fundus assessment. It enables healthcare providers to quickly ascertain the condition of the eye’s fundus before surgery, ensuring it is normal or identifying any preexisting retinal conditions. Additionally, after dense cataract surgery, smartphone fundus imaging can be used for the post-surgical confirmation of the fundus’ condition, which could not be conducted preoperatively due to severe lens opacity (Figure 3). These applications are crucial to ensure the fundus is normal or abnormal before or after surgery in the operating room without the need for a table-top fundus camera, and to check for any postoperative complications such as retinal detachment or hemorrhages. The convenience of smartphone fundus imaging allows for immediate examination in a non-invasive manner, providing reassurance to both the patient and the surgeon. The intraoperative use of smartphone fundus imaging is feasible in cases where aseptic techniques are not strictly required (such as extraocular surgery) or when the imaging can be performed using aseptic techniques. 

#### 4.4.2. Selfie Fundus Imaging

**Figure 4:**
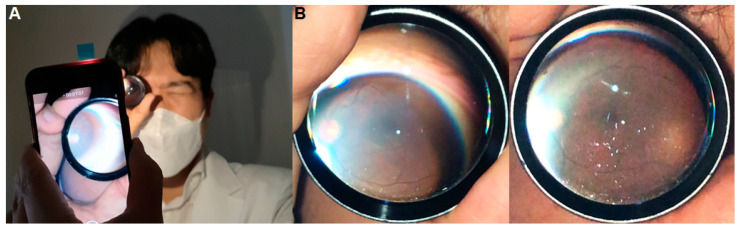
Smartphone “selfie” fundus imaging technique (**A**) and results (**B**).

Smartphone fundus imaging has opened up new possibilities for the self-examination and monitoring of the retina, allowing individuals to capture “selfie” images of their own retina (Figure 4). This application holds significant potential in the field of telemedicine and remote patient monitoring [47].

By using a smartphone camera in conjunction with a condensing lens, individuals can capture high-quality images of their retina without the need for specialized equipment or a trained professional. This empowers patients to actively participate in monitoring their eye health, particularly for those with chronic conditions like DR or AMD. Patients can capture retinal images at regular intervals and share them with their healthcare providers, enabling remote evaluation and timely interventions if any concerning changes are detected. The convenience and accessibility of smartphone fundus imaging promote better adherence to follow-up imaging and facilitate the early detection of potential complications, ultimately improving patient outcomes.

## 5. Future Directions

### 5.1. Technological Advancements and Standardization

Improving image quality and field of view is a key area of focus for the future of smartphone fundus imaging. Advancements in smartphone camera technology, including higher resolution sensors, improved optics, and computational photography techniques, will enable the capture of more detailed and high-quality retinal images. Additionally, the development of specialized lenses and adapters designed specifically for smartphone fundus imaging can expand the field of view, allowing for a more comprehensive examination of the peripheral retina.

The standardization of techniques and devices is another important aspect that will drive the widespread adoption of smartphone fundus imaging. Establishing standardized protocols and guidelines for image acquisition and processing will ensure consistency and reliability across different settings [5]. Furthermore, the development of user-friendly and cost-effective devices tailored for smartphone fundus imaging will make this technology more accessible to a broader range of healthcare providers. With improved image quality and standardized techniques, healthcare professionals will be better equipped to detect and diagnose various retinal conditions accurately. Additionally, the portability and ease of use of smartphone fundus imaging make it an ideal tool for large-scale screening programs, enabling early detection and timely intervention for conditions like DR and AMD. Increased screening efficiency is anticipated as smartphone fundus imaging technology matures.

### 5.2. Integration with Artificial Intelligence and Machine Learning

The use of artificial intelligence (AI) and machine learning for automated disease detection from smartphone fundus images is a promising area of development [48]. AI algorithms can be trained to analyze smartphone-based retinal images and identify patterns associated with various eye diseases, potentially reducing the burden on healthcare professionals and improving diagnostic accuracy. However, developing robust AI algorithms for smartphone fundus imaging presents several challenges [48]. These include the need for large, diverse, and well-annotated datasets for training, as well as addressing potential biases and ensuring the generalizability of the algorithms across smartphone devices, different populations, and imaging conditions. Prediction and guidance to therapy are also potential applications of AI in smartphone fundus imaging [49]. By analyzing retinal images and patient data, AI systems could potentially predict the progression of eye diseases and suggest personalized treatment recommendations [50]. This could lead to more proactive and targeted interventions, improving patient outcomes and reducing the disease burden on healthcare systems and society.

However, the integration of AI into smartphone-based fundus imaging presents several critical considerations, particularly concerning legal responsibilities, computational limitations, and potential conflicts of interest. One significant issue is the legal responsibility when AI-driven diagnostic results are incorrect. Unlike traditional medical examinations performed by certified professionals, AI algorithms operate autonomously, raising questions about accountability. If a diagnosis made by an AI on a smartphone leads to incorrect treatment or harm, it is unclear whether liability should fall on the software developers, the medical institutions utilizing the technology, or the physicians incorporating AI into their practice. This ambiguity necessitates the establishment of clear legal frameworks and guidelines to delineate responsibility and ensure patient safety [41,51]. 

Moreover, the computational limitations of smartphones pose another challenge. Although smartphones are ubiquitous and offer a low-cost alternative for fundus imaging, their limited processing power can result in long inference times for AI algorithms. This can be particularly problematic in emergency settings where rapid diagnosis is crucial. For instance, studies have shown that while smartphone-based devices like the D-Eye can effectively detect ocular abnormalities, the analysis time and image quality can vary significantly [5,41]. Therefore, optimizing AI algorithms for efficiency and ensuring they can operate within the constraints of smartphone hardware are essential for practical implementation. Additionally, this requires balancing the need for high-quality diagnostic images with the processing capabilities of the device, which can be a challenging technical hurdle [3]. 

Lastly, the introduction of AI in smartphone fundus imaging could potentially lead to conflicts of interest between healthcare providers and technology companies. Physicians might be skeptical of integrating AI tools from commercial entities if they perceive these tools as undermining their professional autonomy or if there are financial incentives involved. Conversely, technology companies may prioritize profit over clinical efficacy, leading to suboptimal patient outcomes. Thus, fostering a collaborative environment where both parties work towards common goals, such as improving patient care and ensuring ethical standards, is vital. This collaboration should include transparent communication, shared responsibilities, and continuous education to align the interests of doctors and tech developers [4,51]. In light of this, while the advent of AI in smartphone-based fundus imaging offers promising benefits, it is imperative to address legal, technical, and ethical challenges to ensure its successful and safe implementation in clinical practice.

### 5.3. Regulatory Landscape and Adoption in Healthcare Systems

As smartphone fundus imaging technology continues to evolve, regulatory approvals and guidelines will play a crucial role in its widespread adoption within healthcare systems. Regulatory bodies, such as the Food and Drug Administration (FDA) and their international counterparts, will need to establish guidelines and standards for the development, validation, and use of smartphone fundus imaging devices and software [52]. These guidelines will ensure the safety, efficacy, and quality of the technology, promoting confidence among healthcare providers and patients alike.

The integration of smartphone fundus imaging with electronic health records (EHRs) is another important aspect that will facilitate its adoption in healthcare systems [53]. By enabling the transfer and storage of retinal images within patients’ medical records, healthcare providers can easily access and review these images, enhancing the continuity of care and enabling better-informed decision making. Standardized protocols for image storage, retrieval, and sharing within EHR systems will be essential for the effective utilization of this technology.

### 5.4. Expansion of Clinical Applications and Utility

Smartphone fundus imaging has the potential for wider clinical indications beyond its current applications. As the technology matures and its capabilities expand, it may find utility in the diagnosis and management of various retinal and ophthalmic conditions. For instance, it could be used for certain neurological or other systemic conditions that also manifest in the retina.

The widespread adoption of smartphone fundus imaging in primary care and community settings is another promising prospect. By equipping primary care physicians, community health workers, and other frontline healthcare providers with this technology, we can facilitate -earlier and more frequent screening of patients with potential eye conditions (e.g., DR). This could lead to earlier interventions, improved patient outcomes, and reduced burden on specialized ophthalmology services. Additionally, the portability and affordability of smartphone fundus imaging make it an ideal tool for community-based screening programs, particularly in underserved and remote areas.

Furthermore, the integration of smartphone fundus imaging with telemedicine platforms can revolutionize access to ophthalmic care. The retinal images captured using smartphones can be securely transmitted to ophthalmologists or retinal specialists for remote evaluation and consultation. This approach can bridge the gap between patients and specialized care, particularly in areas with limited access to ophthalmologists. It can also facilitate timely diagnosis and treatment recommendations, improving the overall quality of care.

Overall, the future of smartphone fundus imaging holds immense potential for expanding its clinical utility and widespread adoption, with technological advancements, standardization efforts, and the integration of AI and machine learning poised to revolutionize the field. However, addressing technical challenges related to image quality, standardization, and the development of robust AI algorithms and regulatory challenges; integrating with healthcare systems; and fostering collaboration among stakeholders will be crucial to realizing the full potential of this innovative technology in improving eye care and enhancing patient outcomes globally.

## 6. Conclusions

In conclusion, smartphone fundus imaging represents a transformative advancement in ophthalmic practice, offering a portable, cost-effective, and accessible alternative to traditional fundus imaging modalities. Smartphone fundus imaging has evolved as a feasible solution to the limitations of traditional fundus cameras, overcoming the barriers of cost, portability, and accessibility. Its emergence has been facilitated by advancements in smartphone technology and the development of specialized attachments and accessories. The technique involves a series of steps, including pupil dilation, the preparation of the smartphone, the positioning of optical lenses, image capture, and subsequent review and documentation. Various tips and advanced methods using adaptors have been explored to enhance the quality and versatility of smartphone fundus imaging.

Smartphone fundus imaging holds significant implications for clinical practice, enabling the earlier detection, diagnosis, and monitoring of retinal diseases. Its integration with telemedicine platforms facilitates remote consultations, particularly in underserved areas, and enhances patient engagement and empowerment. In research, smartphone fundus imaging opens avenues for large-scale screening programs, longitudinal studies, and the development of artificial intelligence algorithms for automated disease detection.

The future of smartphone fundus imaging is promising, with ongoing technological advancements aimed at improving image quality, the field of view, and standardization. Integration with artificial intelligence and machine learning will further enhance diagnostic accuracy and screening efficiency. Regulatory considerations and adoption in healthcare systems will be critical in realizing the full potential of smartphone fundus imaging in clinical practice.

Smartphone fundus imaging has the potential to revolutionize patient care by facilitating earlier diagnosis, personalized treatment approaches, and the continuous monitoring of retinal conditions. Its widespread adoption can improve access to essential eye care services, particularly in low-resource and remote settings, thereby reducing the burden of preventable blindness and vision impairment on public health. Further research is warranted to validate the diagnostic accuracy and reliability of smartphone fundus imaging across diverse populations/indications and clinical settings. Additionally, longitudinal studies are needed to assess the effectiveness of smartphone fundus imaging in detecting and monitoring disease progression and treatment response. Standardization efforts and regulatory guidelines should also be developed to ensure the safe and effective integration of smartphone fundus imaging into clinical practice. Through continued research, technological advancements, and collaborative efforts, smartphone fundus imaging can pave the way for improved patient outcomes and enhanced public health impact globally.

## Figures and Tables

**Table 1:** Advantages and limitations of smartphone fundus imaging.

Advantages	Limitations
Accessibility and portability Cost-effectiveness Familiarity and improved patient engagement Continuous improvements and updates by continuous software and hardware improvements	Limited image quality, resolution, and field of view A lack of standardization and calibration leading to variability in image quality and diagnostic accuracy Regulatory issues for medical devices Medicolegal issues such as patient privacy, data security, the risk of unauthorized access, and the potential breaches of confidentiality

**Table 2:** Summary of clinical applications of smartphone fundus imaging for retinal diseases in previous studies.

Clinical Application	Study (Year)	Main Outcome Measures	Findings
Diabetic Retinopathy (DR)	Ryan et al. (2015) [33]	Diagnostic capability for DR detection	Sensitivity: 50%; specificity: 94% (81% and 94%, respectively, using a non-mydriatic fundus camera)
	Russo et al. (2015) [34]	Diagnostic capability for clinically significant macular edema	Sensitivity: 81%; specificity: 98%
	Toy et al. (2016) [6]	Diagnostic capability for moderate nonproliferative and worse DR detection	Sensitivity: 91%; specificity: 99%
	Wintergerst et al. (2020) [11]	Diagnostic capability for DR detection	Sensitivity/specificity: 0.79/0.99 for any DR and 1.0/1.0 for severe DR, 0.79/1.0 for diabetic maculopathy
	Gobbi et al. (2022) [35]	Diagnostic capability for DR detection	Sensitivity: 71%; specificity: 94%
Retinopathy of Prematurity (ROP)	Goyal et al. (2019) [8]	Image quality of wide-field smartphone imaging	Good image quality in 89.3%
	Wintergerst et al. (2019) [12]	Diagnostic capability for ROP and plus disease	Sensitivity: 90% and specificity: 100% for plus disease Sensitivity: 88% and specificity: 93% for ROP
	Patel et al. (2019) [36]	Agreement between the gold standard and photographic assessment of the presence of plus disease	Substantial agreement (Cohen’s kappa = 0.85)
	Lin et al. (2022) [37]	Agreement between binocular indirect ophthalmoscopy and smartphone-based imaging on plus disease	Moderate agreement (Cohen’s kappa = 0.619)
Age-related macular degeneration	Sayadia et al. (2022) [38]	Diagnostic accuracy	From 94.3% to 100% depending on the database used
Hypertensive retinopathy	Sajid et al. (2023) [39]	Diagnostic accuracy	99%

## Data Availability

Not applicable.

## Supplementary Materials

The following supporting information can be downloaded at: https://www.mdpi.com/article/10.3390/diagnostics14131395/s1, Table S1: Summary of content and main focus in key review articles.
