# Supplementary material for: Clinical Applications and Future Directions of Smartphone Fundus Imaging
Source: Diagnostics (Basel). 2024 Jun 30;14(13):1395. doi: 10.3390/diagnostics14131395 (PMC11240943; doi:10.3390/diagnostics14131395)
Supplement: Supplementary file 1 [file diagnostics-14-01395-s001.zip › diagnostics-3038853-supplementary.pdf]

**Table S1**. Summary of the content and main focus in key review articles. Underlined texts indicate the areas where this review differs from the previous ones, adding unique contributions to the literature.

| Reference No. | Authors | Year | Contents | Main focus |
|---|---|---|---|---|
| 10 | Iqbal | 2021 | Optics of smartphone retinal imaging, techniques and devices of smartphone retinal imaging, and safety | Techniques of imaging |
| 3 | Wintergerst et al. | 2020 | An overview of smartphone-based fundus imaging, current applications, and limitations | The current state of smartphone fundus imaging |
| 17 | Prayogo et al. | 2023 | The accuracy of smartphone-based retinal photography for diabetic retinopathy screening | Diabetic retinopathy screening |
| 25 | Hunt et al. | 2021 | Smartphone-based imaging systems for medical use and a critical review of various systems | The methods and materials of smartphone imaging |
| | Ours | 2024 | Advantages and limitations, techniques and tips, clinical applications (including conventional and novel indications), and future directions | Clinical applications including conventional and novel indications |
